# Adapting Tension-Type Headache to the International Association for the Study of Pain (IASP) Taxonomy: A Conceptual Proposal

**DOI:** 10.3390/biomedicines14092058

**Published:** 2026-09-13

**Authors:** Margarita Cigarán-Mendez, Juan C. Pacho-Hernández, Angela Tejera-Alonso, Cristina Gómez-Calero, Lars Arendt-Nielsen, Jo Nijs, César Fernández-de-las-Peñas

**Affiliations:** 1 Department of Psychology, Universidad Rey Juan Carlos, 28922 Alcorcón, Spain; margarita.cigaran@urjc.es (M.C.-M.); angela.tejera@urjc.es (A.T.-A.); 2 Department of Research and Psychology in Education, Universidad Complutense de Madrid, 28040 Madrid, Spain; jpacho@ucm.es; 3 Department Physical Therapy, Occupational Therapy, Physical Medicine and Rehabilitation, University Rey Juan Carlos, 28922 Alcorcón, Spain; cristina.gomez@urjc.es; 4 Center for Neuroplasticity and Pain (CNAP), Sensory-Motor Interaction (SMI) Center, Department of Health Science and Technology, Faculty of Medicine, Aalborg University, DK-9220 Aalborg, Denmark; lan@hst.aau.dk; 5 Department of Gastroenterology & Hepatology, Mech-Sense, Clinical Institute, Aalborg University Hospital, DK-9000 Aalborg, Denmark; 6 Steno Diabetes Center North Denmark, Clinical Institute, Aalborg University Hospital, DK-9000 Aalborg, Denmark; 7 Pain in Motion Research Group (PAIN), Department of Physiotherapy, Human Physiology and Anatomy, Faculty of Physical Education & Physiotherapy, Vrije Universiteit Brussel, BE-1090 Brussels, Belgium; jo.nijs@vub.be; 8 Unit of Physiotherapy, Department of Health and Rehabilitation, Institute of Neuroscience and Physiology, Sahlgrenska Academy, University of Gothenburg, SE-405 30 Gothenburg, Sweden

**Keywords:** tension-type headache, nociceptive, neuropathic, nociplastic, phenotyping

## Abstract

Tension-type headache (TTH) is a primary headache characterized by treatment effect heterogeneity, possibly reflecting variations in underlying pain mechanisms. Identifying pain phenotyping or classifying patients into subgroups based on their predominant pain mechanism may provide targeted treatment options. This paper proposes a conceptual adaptation of the International Association for the Study of Pain (IASP) clinical criteria to identify the predominant phenotype in individuals with TTH. It has been found that pain referrals elicited by trigger points (TrPs) reproduce headache symptoms in patients with TTH. Since preliminary studies suggest that TrPs can represent a peripheral nociceptive input, this could lead to considering TTH as a potential nociceptive condition. However, TrP treatment has limited short-term effects for reducing headache frequency, intensity or duration, requiring us to look beyond this possible peripheral driver. Thus, patients with TTH exhibit altered nociceptive processing, which is compatible with nociplastic pain features. Arguably, treatment approaches for nociplastic phenotypes should address maladaptive factors associated with altered nociceptive processing. Based on the current conceptual proposal, TTH should not be viewed as a homogeneous condition but rather as a spectrum in which nociceptive and nociplastic phenotypes coexist with different predominance according to the disease stage and severity. Collectively, it seems that nociceptive and nociplastic mechanisms may contribute to TTH across its clinical spectrum, with nociplastic features appearing to become more prominent in patients with the chronic form of headache. Recognizing the coexistence of nociceptive and nociplastic pain mechanisms may facilitate precision pain management and improve treatment selection in phenotype patients with TTH. In either case, future trials should identify treatment options with long-term effects for TTH, as currently used approaches only yield short-term benefits.

## 1. Introduction

Headache disorders, particularly migraine and tension-type headache (TTH), represent the second leading cause of years lived with disability (YLD) worldwide [1]. In fact, TTH is probably the most prevalent primary headache. Although prevalence estimates are heterogeneous depending on the methodology and population, it has been estimated that TTH has a point prevalence of 42% [2] and a one-year prevalence of 21% [3] in the general population. It has been recently found that TTH exhibits a global age-standardized prevalence of 24.9% worldwide [4].

There is a debate about peripheral or central drivers in TTH [5]. Available data supports that TTH pathogenesis involves both central and peripheral mechanisms, but the precipitating mechanisms of a headache attack are still not clear. Current theories claim for a peripheral origin of a first tension-type headache attack, which, if repeated and prolonged in time (temporal or spatial summation) could lead to hyperexcitability of the central nervous system favoring the evolution from the episodic to a chronic form of TTH [5].

The diagnosis of TTH is performed following the International Classification of Headache Disorders criteria (ICHD-III) [6]. The ICHD-III includes the following clinical pain features: bilateral location, pressing/tightening pain quality, moderate intensity, no aggravation of headache with physical activity, and no more than one associated sign, e.g., photophobia, phonophobia or mild (no severe) nausea and no vomiting [6]. Thus, TTH is classified according to the frequency of headache as infrequent episodic tension-type headache (IETTH, code 2.1, <1 attack/month), frequent episodic tension-type headache (FETTH, code 2.2, 1–14 attacks/month), and chronic tension-type headache (CTTH, code 2.3, ≥15 attacks per month) [6]. Finally, the ICHD-III subgroups TTH associated with (code 2.1.1, 2.2.1, 2.3.1) or not associated with (code 2.1.2, 2.2.2, 2.3.2) tenderness [6].

The taxonomy of the International Association for the Study of Pain (IASP) considers three phenotypes: nociceptive, neuropathic, and nociplastic pain [7]. The term nociplastic was defined as “pain that arises from altered nociception despite no clear evidence of actual or threatened tissue damage causing the activation of peripheral nociceptors or evidence for disease or lesion of the somatosensory system causing pain” [8]. Although this definition was initially well-accepted, it has also raised several questions [9]. The first situation is that discrimination between these three phenotypes can be found to be challenging for clinicians, since patients can fit into more than one phenotype (e.g., mixed-type pain), since one phenotype does not exclude another one and the same pain condition can evolve from one phenotype to another [10]. Second, clinical identification of altered nociceptive pain processing is highly difficult since no gold standard exists for classifying heightened pain responses and no standardized tools for clinical practice exist [11].

In the last years, there have been attempts to adapt the nomenclature of the headache literature to IASP taxonomy. Cropes et al. proposed that cervicogenic headache could exhibit two predominant pain phenotypes, nociceptive or nociplastic [12]. May et al. found that persistent idiopathic facial pain also exhibits brainstem changes compatible with a nociplastic pain condition [13]. The application of IASP taxonomy to primary headaches, such as TTH, has not been previously investigated.

In 2021, the IASP proposed a set of clinical criteria as a grading system for orientating the identification of pain phenotypes [14]. Identification of the predominant pain phenotype has the potential to improve precision pain medicine practice since, presumably, patients with pain will respond best to interventions that most effectively target their predominant mechanism [15]. In fact, distinction between pain phenotypes seems to be crucial because nociplastic phenotypes can be more difficult to treat than other pain phenotypes. The IASP clinical criteria were originally developed to be applied to chronic nociplastic conditions affecting the musculoskeletal system [14]. It seems that these criteria could be also applied to primary headaches such as TTH. The current paper proposes a conceptual adaptation of the IASP clinical criteria to identify the predominant phenotype in individuals with TTH.

## 2. Phenotyping Tension-Type Headache as Nociceptive Pain

Nociceptive pain is defined as “pain attributable to the activation of the peripheral receptive terminals of primary afferent neurons, particularly of non-neural tissues, in response to different noxious stimuli, and clinically, the pain response is proportional to the nociceptive input” [16]. It could be hypothesized that headache features experienced in people with TTH could be attributable to a predominant nociceptive mechanism, but direct evidence supporting this notion is limited. Headaches are described as pressing, tightening, dull, heaviness and soreness [6]; features like descriptors used for featuring muscle pain. Muscle pain is associated with the activation of skeletal peripheral nociceptors by the release of a variety of inflammatory mediators, e.g., bradykinin (BK), serotonin (5-HT), calcitonin gene-related peptide (CGRP), nerve growth factor (NGF), interleukins (IL-6, IL-8), substance P, or tumor necrosis factor-α (TNF-α) [17]. However, the presence of such pro-inflammatory factors suggests a response as part of a condition, unhealthy lifestyle, or ongoing tissue (muscle) damage, and should not be interpreted as a feature of muscle pain per se. In an experimental model, hypertonic saline injection into splenius capitis, upper trapezius or temporalis muscles was able to elicit referred pain perceived as headache mimicking TTH in healthy people [18]. Such observations created the idea that TTH might be partly related to muscle tissue dysfunctions, which might serve as a nociceptive source in people with TTH [19,20].

From a clinical point of view, TTH has been associated with myofascial trigger points (TrPs). Trigger points are defined as “hypersensitive spots located into taut bands of skeletal muscles that are painful upon stimulation, which usually feature referred pain sensations and associated phenomena” [21]. Clinical identification of TrPs is mainly based on manual palpation and the presence of associated referred pain [22], but no quantitative, objective, or assessment criteria exist, leaving us wondering exactly what type of nociceptive source they are. However, the presence of muscle TrP-associated referred pain in people with TTH is supported in the literature [23,24]. In fact, several studies have observed that referred pain elicited by TrPs in the cervical and shoulder muscles reproduces the pain features of headache attacks in individuals with TTH [23,24]. From a treatment viewpoint, in nociceptive pain conditions, functional activity and early treatments targeting the nociceptive input are proposed as the therapeutic target. If we consider the presence of TrPs as a potential source of nociception, damping the peripheral nociceptive driving may not only reduce the number of attacks but may prevent, or also delay, the transition from episodic into chronic headaches. Thus, TrP treatment has been found to decrease pain sensitivity in the short-term in people with TrP-associated referred pain, but there is no support for long-term effects [25,26]. Further, manual treatment targeting TrPs has been found to be effective for reducing the frequency, intensity and duration of headaches for the short-term in individuals with TTH [27]. In summary, evidence supporting the treatment of TTH as a predominant nociceptive condition is limited and even non-existing when taking criteria for high-value care (long-term effects) into account. Hence, we need to look beyond nociceptive muscle pain in TTH.

Thus, the evidence favoring TTH just as a nociceptive pain condition is questionable since identification of the source of nociception (muscle-associated referred pain) is not always possible [28]. This does not exclude the possibility of individual patients presenting predominant nociceptive TTH (particularly in the episodic form), but at least it suggests that we need to look beyond nociceptive pain in individuals with TTH and should also consider other predominant phenotypes such as nociplastic pain, and at the same time conduct rigorous studies identifying other possible sources of nociceptive pain in TTH. In fact, Pensri et al. have recently identified two clusters of individuals with TTH, one without sensitivity and another one with high pain sensitivity [29]. These authors observed that the presence of active TrPs (whose associated referred pain reproduce the headache attacks) was mostly present in the high-sensitive cluster, which was associated with longer headache history, more painful bodily areas, higher tenderness score and more symptoms on musculoskeletal testing [30].

## 3. Phenotyping Tension-Type Headache as Neuropathic Pain

According to the Neuropathic Pain Special Interest Group (NeuPSIG) of the IASP, the pain of neuropathic origin is defined if: (1) there is lesion/disease of the somatosensory nervous system, either peripheral or central nervous system; (2) symptoms are limited to a neuroanatomically distribution plausible with affectation of the nervous system; and (3) pain is supported by examination findings as well as laboratory and/or imaging [31]. It seems clear that primary headaches such as migraine or TTH should not be classified as neuropathic-like pain in nature, since no evidence of nervous system damage is present. However, increasing evidence suggests that some pain conditions traditionally considered not to be neuropathic can exhibit morphological evidence of sensory nervous system pathology such as small nerve fiber pathology. A recent meta-analysis observed that some non-neuropathic chronic pain conditions, e.g., fibromyalgia or irritable bowel syndrome, showed evidence of small nerve fiber pathology [32]. A subgroup analysis of studies including individuals with migraine found that this primary headache is not associated with nerve damage [32]. No data on TTH and small nerve fiber pathology is available.

## 4. Phenotyping Tension-Type Headache as Nociplastic Pain

According to the IASP definition of nociplastic pain, sensitization of the central nervous system (e.g., increased responsiveness of nociceptive pain neurons within the central nervous system to their normal or subthreshold afferent input [33]) is thought to be the underlying mechanism of this phenotype, although it cannot be measured directly in humans but only by surrogate measures [9].

Patients with TTH exhibit altered pain processing, although there is a debate if this altered pain processing is present in both forms, episodic and chronic, or just within the chronic form [5,34]. It has been found that people with TTH exhibit pain response hyperexcitability at spinal (trigeminal) and supraspinal (brainstem) levels [35,36,37]. In fact, moderate evidence supports the presence of trigeminal hyperalgesia to pressure pain (spinal sensitization) in both episodic/chronic TTH and the presence of widespread hyperalgesia to pressure pain (supraspinal sensitization) just in the chronic form [38]. In addition, temporal summation and impaired conditioned pain modulation had been also found in people with CTTH [39,40,41]. Nevertheless, it is possible that not all individuals with CTTH exhibit all features of the altered pain processing. Exposto et al. identified different pronociceptive pain profiles in individuals with CTTH that could be associated with an increased temporal summation, a deficient conditioned pain modulation or both [42]. The debate is placed on the degree of altered pain processing in the episodic form. It is probable that peripheral and central mechanisms are involved at the same time in both forms leading to a mixed-type pain or a modifiable pain phenotype. This theory is supported by the fact that changes observed in brainstem regions involved in pain processing (e.g., anterior cingulate cortex, supramarginal gyrus, temporal pole, lateral occipital cortex and caudate) in people with CTTH [43,44] are reversible and even absent during headache-free periods in people with the episodic form [45]. Other authors proposed the hypothesis that episodic and chronic TTH represents a continuum pain spectrum [46]. Collectively, we hypothesize that nociceptive and nociplastic mechanisms may contribute to TTH across its clinical spectrum, with nociplastic features being more prominent in CTTH.

Another clinical finding supporting the presence of altered pain processing in TTH is the presence of widespread pain symptomatology. In fact, up to 80% of patients with TTH report symptoms in extra-cranial areas, e.g., neck, upper or lower back [47,48,49]. Thus, the presence of extra-cranial pain in individuals with chronic headaches was associated with widespread pressure pain sensitivity [50].

Other central nervous system-derived symptoms, such as fatigue, poor sleep quality, or mood disturbances, related to neuro-immune alteration are also typical of nociplastic conditions [51], and have also been seen in individuals with TTH [52,53]. A meta-analysis recently found moderate evidence supporting a positive association between depression/anxiety with headache frequency [54]. Thus, psychological factors affect pain processing throughout the central nervous system by aggravating sensitivity to pain [55]. It is interesting to note that stress increases hypersensitivity responses to pressure pain in TTH [56] and that stress is one of the main triggering factors for a headache attack. This association is supported by a meta-analysis showing that anxiety and stress are potential predictors of TTH chronification [57].

At this point, the presence of altered pain processing in patients with TTH is compatible with nociplastic features. It seems that both nociceptive and nociplastic pain phenotypes can coexist with more/less predominance in the same patient with TTH. Therefore, it seems that TTH should not be interpreted as a homogeneous entity but rather as a complex condition resulting from a dynamic interaction between peripheral mechanism, central processes, and contextual/behavioral modulators.

## 5. Clinical Criteria System for Phenotyping of Tension-Type Headache

This section will adapt the original IASP clinical criteria [14] to TTH for determining the predominant phenotype in this primary headache. Clinicians should consider that one patient can fulfill criteria for more than one phenotype (mixed-type phenotype), and the same patient can evolve to another phenotype; therefore, the aim should be to first determine which pain phenotype is the predominant in a particular patient at a specific moment.

### 5.1. Step 1: Duration of Pain

The classical requirement for chronic pain is that symptoms must be present for at least 3 months. In the headache literature, chronic headaches are defined according to the frequency of the attacks and not according to the history with pain [6]. For collecting headache features, a 12-week diary is usually recommended [58,59]. Accordingly, subjects suffering from headaches for more than three months will fulfill step one, independently of the frequency or attacks.

### 5.2. Step 2: Distribution of Pain

A nociceptive pain pattern is usually localized, with neuroanatomical sense, and is exacerbated with specific pain triggers; a neuropathic pain pattern usually follows the anatomical trajectory of a peripheral nerve, whereas a nociplastic pain pattern is more generalized and/or widespread [14]. Patients with TTH commonly perceive pain around all their head during their headache attacks, the frontal, suboccipital, and temporal areas being the most affected [60].

It is important to note that several patients with TTH experience pain in extra-cranial pain areas [46,47,48]. Further, it has been observed that almost 30% of individuals with TTH also fulfill the criteria of fibromyalgia syndrome [61,62], a condition currently considered as a nociplastic phenotype [63,64]. Accordingly, a careful assessment of the pain pattern of a patient with TTH during clinical assessment is needed to identify changes and the evolution of the symptoms from a localized (cranial) to a more generalized (extra-cranial) pattern. Specific pain drawings can be used to standardize and optimize clinical assessment of the individual’s pain distribution in a reliable and valid way [65].

### 5.3. Step 3: Determine Whether Nociceptive Pain Is Present

The next step is to identify if the pain pattern can be entirely explained by nociceptive mechanisms [14]. If we consider the presence of TrPs as a nociceptive pain condition [66], headache attacks reproduced by TrP-related referred pain could be considered a sign of nociceptive pain. Still, such a clinical observation should always be interpreted considering the entire clinical picture (i.e., such TrP-elicited headache attacks can only be a sign of nociceptive pain in the absence of widespread pain, without or with very few other signs of central nervous system hypersensitivity). Some studies have identified TrPs as potential pain nociceptive generators using imaging technique [67] and specific biomarkers [68]. Thus, it is important to consider that the identification of a nociceptive source does not exclude a concomitant nociplastic mechanism.

### 5.4. Step 4: Determine Whether Neuropathic Pain Is Present

A mandatory criterion for nociplastic pain states that symptoms cannot entirely be explained by a neuropathic pain mechanism [14]. It is almost unlikely that a patient with TTH exhibits somatosensory nervous system damage [32].

### 5.5. Step 5: Elucidate the Presence of Pain Hypersensitivity

The fifth step involves screening for signs of hypersensitivity (i.e., hyperalgesic or allodynic responses) [14]. Nociceptive pain is characterized by primary hyperalgesia (i.e., hypersensitivity in the painful area), whereas nociplastic pain is characterized by secondary hyperalgesia (i.e., hypersensitivity within distant pain-free areas). Several studies have seen that patients with TTH exhibit hyperalgesia to pressure pain within trigeminal (primary hyperalgesia), extra-trigeminal and/or distant pain-free (secondary hyperalgesia) areas [38]. Nevertheless, meta-analytic data found that the presence of trigeminal and extra-trigeminal pain hyperalgesia was just present in the chronic form, whereas the episodic form exhibited hypersensitivity just within the trigeminal area [38]. According to the IASP clinical criteria, at this stage a patient fulfilling this criterion can be classified as having “possible nociplastic pain” [14].

### 5.6. Step 6: Check for History of Pain Hypersensitivity

Step 6 involves examining whether the patient reports symptoms of hypersensitivity, particularly allodynic responses. Cutaneous allodynia is formerly reported by up to 60% of patients with migraine [69], but its presence in TTH has not been properly investigated. One study reported that 30% of individuals with severe episodic TTH reported cutaneous allodynia [70]. If a patient meets Step 6, then pain can be considered “probably nociplastic phenotype” [14].

### 5.7. Step 7: Determine Whether Comorbid Symptoms Are Present

The final step involves screening for the presence of (1) hypersensitivity to different stimuli, e.g., sound (phonophobia), light (photophobia); (2) a comorbid medical condition; (3) other nervous system-associated symptomatology, including sleep quality, fatigue and cognitive problems [14]. First, according to the ICHD-III, the presence of phonophobia and photophobia is a cardinal sign of migraine diagnosis, although in patients with CTTH one of these associated symptoms can also be present [6]. Identifying whether a patient with TTH also has migraine is a challenging process in clinical practice, since TTH and migraine share common features, but there is a lack of specific diagnostic tests and biomarkers supporting a differential diagnosis [71]. Second, one third of individuals with TTH have comorbid fibromyalgia, a condition with nociplastic features [61,62]. In fact, TTH is included in the term of “chronic overlapping pain conditions”, a set of pain conditions (e.g., irritable bowel syndrome, interstitial cystitis, temporomandibular pain, chronic fatigue syndrome, endometriosis) that are frequently comorbid [72]. Third, the presence of other associated symptoms, e.g., fatigue, anxiety/depression, poor sleep quality, are also commonly observed in people with TTH [52,53].

**Figure 1:**
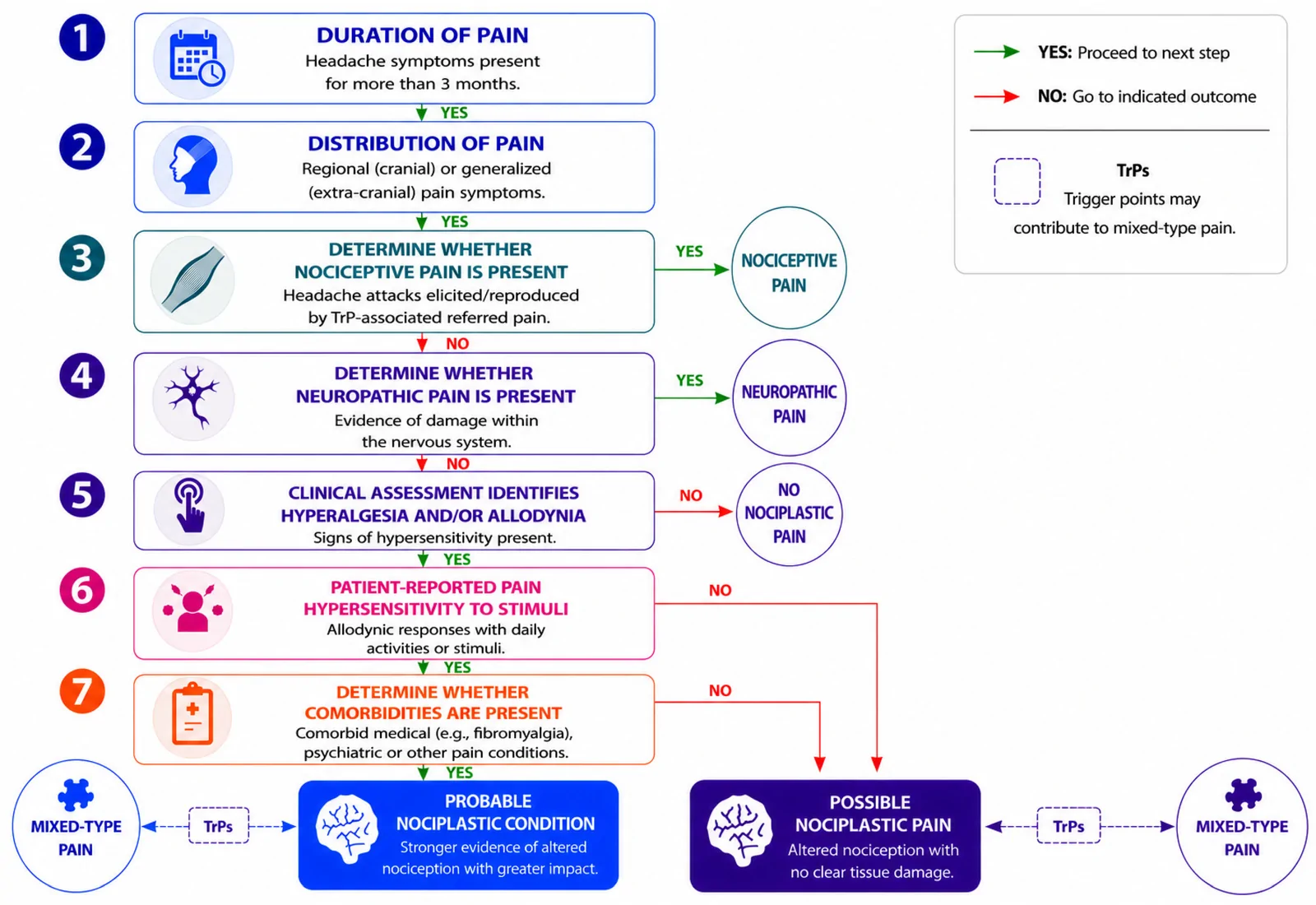
Clinical decision-making tree for identifying the pain phenotype of patients with tension-type headache (TTH). Adapted from 2021 IASP clinical criteria [14].

If all criteria are fulfilled, TTH should be classified as “probable nociplastic pain” [14]. Figure 1 provides a proposed conceptual adaptation of the clinical decision-making tree for clinicians based on the IASP criteria for assessing the predominant pain phenotype in subjects with TTH. Despite positive findings from early reliability testing using clinical vignettes of patients with musculoskeletal pain conditions [73], it is important to note that more research is needed to examine the reliability and validity of the 2021 IASP clinical grading criteria in specific pain conditions [14]. Studies examining the reliability and validity of the IASP clinical grading criteria in patients with TTH, as presented in this paper, are lacking. Further, more research is needed to determine the prognostic value and responsiveness of these criteria for the improvement of treatment outcomes in clinical trials.

## 6. Considering Pain Phenotype in Tension-Type Headache Treatment

This conceptional proposal suggests that, due to heterogeneous pathophysiology, patients with TTH should be managed according to their underlying pathophysiology. Thus, the degree of nociplastic phenotype in individuals with TTH could be inversely related to the clinical improvement of isolated peripherally based treatments; therefore, the identification of individuals with TTH with nociplastic features can be relevant for determining more appropriate therapeutic approaches. Accordingly, when developing a treatment plan, determining the predominant phenotype should be included in the clinical decision tree. It should be noted that no study has investigated and demonstrated that phenotyping predicts treatment response in patients with TTH.

### 6.1. Pharmacological Treatment of Tension-Type Headache

Current management of TTH involves pharmacological and non-pharmacological interventions. In addition, pharmacological treatment can be divided into symptomatic (medication taken for a headache attack) and prophylactic (prolonged medication taken used for preventing headaches). Analgesics, i.e., paracetamol, and non-steroidal anti-inflammatory drugs (NSAIDS), i.e., ibuprofen, are considered as the first-line symptomatic treatment choice by the European Federation of Neurological Societies (EFNS) and the U.S. Department of Veterans Affairs/U.S. Department of Defense Clinical Practice Guidelines for managing TTH [74,75]. This recommendation agrees with Cochrane reviews reporting moderate to high evidence for the use of paracetamol (1000 mg) [76] or ibuprofen (400 mg) [77] as effective acute medications for TTH, particularly for the episodic form. A recent network meta-analysis has confirmed ibuprofen as the most effective acute medication and diclofenac-K as the second one for ETTH [78]. Fernández-de-las-Peñas et al. identified that the early taking of acute medication during an attack may be protective and can prevent further chronification [79]. Considering the peripheral analgesic action of NSAIDs and the central action of analgesics, these medications may be relevant to the management of both nociceptive and nociplastic TTH phenotypes; however, this hypothesis has not been tested and should be demonstrated by phenotype-stratified TTH trials.

Amitriptyline is the first-line prophylactic treatment option proposed by the EFNS and U.S. Department of Veterans Affairs/U.S. Department of Defense Clinical Practice Guidelines for managing CTTH [74,75]. This recommendation has been supported by a recent network meta-analysis showing amitriptyline as the highest ranked treatment for reducing headache frequency in individuals with CTTH [80]. It is important to note that prophylactic medication (mainly antidepressants) led to a reduction in headaches due to a potential anti-nociceptive effect rather than to an antidepressant effect, related to the low dose (50–100 mg) recommended for headache sufferers. An effect on the central nervous system by antidepressants would be more relevant for patients with nociplastic features, although the exact mechanisms behind amitriptyline’s benefit for TTH is not definitively established. Botulinum toxin A (BTX-A) is a neurotoxin that is commonly applied to treat different headaches, but its scientific evidence has been questioned. Dhanasekara et al. found that the application of BTX-A was associated with clinical improvements for the intensity and frequency of headache and acute pain medication used in individuals with CTTH [81]. It is suggested that BTX-A acts at both peripheral and central pain regulatory mechanisms [82]. Thus, BTX-A is also a potent skeletal muscle relaxant and has been also applied to treat myofascial TrPs, although evidence is low to moderate [83].

It could be hypothesized that pharmacological treatment commonly used for TTH can act on nociceptive and nociplastic TTH phenotypes; however, both acute and preventive medications only act partially on the peripheral nociceptive drive and the central nervous system; therefore, proper clinical identification of peripheral and central nervous drivers is important [84]. Future clinical trials evaluating the predictive ability of identifying the pain phenotype in individuals with TTH are needed.

### 6.2. Non-Pharmacological Treatment of Tension-Type Headache

Non-pharmacological therapies are included in most international guidelines related to the management of TTH. The EFNS Clinical Practice Guideline suggests that non-pharmacologic therapies should be considered for managing TTH [74]. Similarly, the Italian guideline for primary headaches also includes non-pharmacological approaches as complementary treatment for patients with headaches, particularly in those individuals where medication may be not a good strategy option [85].

Different meta-analyses have observed that physical therapy interventions can be effective for managing TTH; however, the results depend on the specific approach. Jung et al. reported that manual therapies combined with exercise or transcutaneous electrical stimulation were effective in reducing the intensity and frequency of headaches in people with TTH [86]. Krøll et al. also concluded that manual therapy and exercise were effective for reducing headache features [87] and improving quality of life [88] of people with TTH. Mesa-Jiménez et al. concluded that manual therapy approaches were more effective than medication for reducing frequency, intensity and duration of the headache in TTH [89]. All meta-analyses found that the effects of these interventions were short-term not long-term, probably because most interventions investigated in these meta-analyses mainly targeted nociceptive pain. As has been proposed in this paper, in individuals with TTH, a potential nociceptive peripheral drive presumably could be the presence of TrPs. It has been found that myofascial TrP interventions led to significant reductions in headache intensity and headache-related disability in people with TTH, but, again, only for the short term [90].

A potential hypothesis explaining these short-lasting effects of manual therapy approaches could be related to the lack of identification of individuals with TTH with a nociplastic phenotype. Accordingly, treatments targeting the nociceptive drive alone should be considered as complementary approaches alongside those treatments targeting the underlying nociplastic mechanism. Hence, it could be arguable that subjects with predominant nociplastic pain should receive treatment approaches targeting those factors that perpetuate and interact with hyperexcitability of the nervous system, e.g., sleep problems, pain hypervigilance, anxiety/depression, stress intolerance, or kinesiophobia. In people with a mixed-type TTH phenotype, a multimodal plan, including treatment of perpetuating and promoting TrP factors, could be needed since removing the peripheral drive (if identified) potentially will have little effect on the central nervous system. This multimodal treatment plan should combine pharmacological treatment and physical therapy, as well as psychological/cognitive behavioral approach. Although psychological interventions have shown to be effective for reducing headache frequency and headache intensity in TTH, the results of these interventions when applied in isolation were just short-term [91]. Thus, more emphasis should be given to integrating stress management as a component of multimodal approaches for managing TTH, as it was shown to be effective (long-term) in individuals with chronic whiplash-associated disorder [92], a nociplastic condition that often includes headaches. In such scenario, clinical management of patients with TTH needs to extend beyond peripheral drive (bottom-up interventions) to incorporate treatment strategies directed at normalizing altered nociceptive processing (top-down interventions). Accordingly, clinical identification of peripheral or central drivers is important [84].

Additionally, the objective of managing individuals with nociplastic pain is that they can develop strategies to optimize functional movement and undertake active exercise. Thus, local exercises targeting the neck/shoulder region have been shown to be effective in reducing the intensity, duration, and frequency of headaches in people with CTTH [93]. However, we do not currently know which type of exercise, e.g., localized or aerobic, resistance or strengthening, is more appropriate for patients with TTH. Underlying pain phenotypes could be considered to optimize exercise prescription, mostly in people with a nociplastic phenotype [94]. In a patient with a nociplastic phenotype, exercise should be applied in a pain-contingent graded way [95] and pacing/cognitive approaches could be also applied in isolation or combined with exercise. No study has investigated this hypothesis in patients with TTH.

Finally, the role of diet and the gut microbiome is becoming increasingly recognized in the chronic pain field [96,97]. Interestingly, a pilot study found that higher intakes of saturated fatty acid and Omega-6 (ω-6) fatty acids were associated with more severe headaches in patients with TTH [98]. Together, this calls for more research exploring the possible role of unhealthy diet and related gut dysbiosis as part of the multiprofessional management of TTH.

## 7. Conclusions

This paper proposes a conceptual adaptation of the IASP clinical decision-making tree to be applied to phenotyping subjects with TTH into nociceptive, nociplastic or mixed-type pain phenotype. It can be hypothesized that TTH should not be viewed as a homogeneous pain condition but rather as a complex condition in which nociceptive and nociplastic pain phenotypes coexist with different predominance according to the stage and individual feature. Collectively, it seems that nociceptive and nociplastic mechanisms may contribute to TTH across its clinical spectrum, with nociplastic features appearing to become more prominent in patients with chronic tension-type headaches. Recognizing the coexistence of nociceptive and nociplastic pain mechanisms may provide new tools to develop precision pain management regimes and thereby improve treatment modalities for patients with TTH. This paper also proposes a conceptual application of this clinical rationale for choosing treatment plans for an individual with TTH based on a predominant pain phenotype, although future clinical trials should confirm the hypotheses proposed.

## Data Availability

No new data were created or analyzed in this study. Data sharing is not applicable to this article.
